# The Epidemiology of Functional Gastrointestinal Disorders in Mexico: A Population-Based Study

**DOI:** 10.1155/2012/606174

**Published:** 2012-03-19

**Authors:** Aurelio López-Colombo, Douglas Morgan, Dalia Bravo-González, Alvaro Montiel-Jarquín, Socorro Méndez-Martínez, Max Schmulson

**Affiliations:** ^1^Puebla Research Coordination, Instituto Mexicano del Seguro Social, CP 72000 Puebla-PUE, Mexico; ^2^School of Medicine, University of North Carolina at Chapel Hill, Chapel Hill, NC 27599-7080, USA; ^3^Hospital General Regional Número 36, Instituto Mexicano del Seguro Social, CP 72090 Puebla-PUE, Mexico; ^4^Laboratory of Liver, Pancreas and Motility (HIPAM), Department of Experimental Medicine, Faculty of Medicine, Hospital General de México, Universidad Nacional Autónoma de México (UNAM), CP 06726 Mexico City, Mexico

## Abstract

*Aims.* The frequency of functional gastrointestinal disorders (FGIDs) in the general population of Mexico is unknown. *Methods.* To determine the prevalence of FGIDs, associated depression, and health care utilization, a population-based sampling strategy was used to select 500 households in the State of Tlaxcala, in central Mexico. Household interviews were conducted by two trained physicians using the Rome II Modular Questionnaire, a health-care and medication used questionnaire and the CES-D depression scale. *Results.* The most common FGIDs were IBS: 16.0% (95% CI: 12.9–19.5); functional bloating: 10.8% (8.2–13.9); unspecified functional bowel disorder: 10.6% (8.0–13.6); and functional constipation (FC): 7.4% (5.3–10.1). Uninvestigated heartburn was common: 19.6% (16.2–23.4). All FGIDs were equally prevalent among both genders, except for IBS (*P* = 0.001), IBS-C (*P* < 0.001), IBS-A/M (*P* = 0.049), and FC (*P* = 0.039) which were more frequent in women. Subjects with FGIDs reported higher frequencies of medical visits: 34.6 versus 16.8%; use of medications: 40.7 versus 21.6%; (both *P* < 0.001); and reported depression: 26.7 versus 6.7%, (*P* < 0.001). *Conclusion.* In this first population-based study of FGIDs in Mexico, heartburn, IBS, functional distension, and FC were common. Only IBS, IBS-C, IBS-A/M, and FC were more frequent in women. Finally, FGIDs in Mexico had an increased burden of health care utilization and depression.

## 1. Introduction

Functional gastrointestinal disorders (FGIDs) are very common and their global impact is often underestimated [1, 2] due to their limited associated mortality [3]. However, it is well documented that FGIDs have a negative impact on health-related quality of life (HRQOL) and have a burden of illness because of the number of physician visits, diagnostic tests, and secondary economic losses due to work absenteeism [4].

In Latin America, there are few studies that have estimated the prevalence and burden of FGIDs and the majority have been conducted in selected populations [5–7]. In Mexico, for example, only one study to date has evaluated the prevalence of all FGIDs using the Rome II Modular Questionnaire (RIIQ) but focused on a University population from Mexico City [8]. This study found that irritable bowel syndrome (FC) (35%), uninvestigated heartburn (35%), functional abdominal bloating, (21%) and functional constipation (FC) (19%) were frequent. Interestingly, uninvestigated dyspepsia was less common (8.0%) [8]. Also, IBS with diarrhea predominance (IBS-D) was less frequent compared to IBS with constipation (IBS-C): 4.6 versus 14.7% and the latter was four times more common in women than men [8]. A second study in Mexico in patients with IBS nationally cared for in private practice, confirmed the lower prevalence of IBS-D compared to IBS-C and although all IBS subtypes showed a female predominance, the percentage of men among those with IBS-D was 1.7 to 2.4 higher than in the other subtypes [9]. Recently, our group reported the analysis of a RIIQ database, noting that the proportion of women among those fulfilling criteria for IBS and uninvestigated dyspepsia (67.8 y 85.4%, resp.,) was significantly higher than the proportion of women among the group not fulfilling criteria for any FGID (55.9%) [10]. In addition, compared to men with IBS, women reported more frequently symptoms related to constipation and abdominal distension [10].

We conducted a population-based study of FGIDs in Mexico, to our knowledge, the first such investigation. Based on our previous study among volunteers in Mexico [8], we hypothesized that uninvestigated heartburn, IBS, FC and uninvestigated dyspepsia would be the most common FGIDs and that all of these disorders together with IBS-C would be more common in women, while IBS-D would be more common in men. In addition, we included assessments of health care utilization and psychological burden.

## 2. Methods

From June 1st to October 31st, 2005, a population-based, cross-sectional study was conducted in the State of Tlaxcala, in central Mexico. In the 2000 population registry (census) of the *Instituto Nacional de Estadística y Geografía (INEGI) *[National Institute of Statistics and Geography], Tlaxcala had 962,646 inhabitants distributed in 60 cities/villages [11]. Anticipating an IBS prevalence ranging between 10 to 20% based on a systematic review of population-based studies in North America that did not include Mexico [12], considering a 10% precision of the outcome factor with a 99.99% confidence interval and a design effect of 1, a sample of 243 subjects was estimated. Therefore, we decided to survey a sample double that size. A population-based sample strategy was used to select 500 subjects representing approximately 0.05% of the State's population. The interviews were conducted in 500 randomly selected households and the number of subjects surveyed within each city/village was proportional to the number of their population. Exclusion criteria included pregnancy and major medical illness at the moment of the survey and a history of gastrointestinal surgery and/or significant psychiatric disease. Per protocol, if the first adult appearing in the household could not be interviewed due to exclusion criteria or recruitment failure, the neighboring household was selected.

Household interviews were conducted by two trained physicians. Demographic information included age, gender, occupation, and marital status. The RIIQ was used to assess abdominal symptoms and diagnose the FGIDs by the Rome II criteria [13]. The RIIQ had been previously translated and validated in Mexico and allowed us to determine the presence of all FGIDs [8]. We acknowledge that the RIIQ identifies uninvestigated heartburn including functional heartburn and gastroesophageal reflux (GER) [14]. The Rome II criteria require a medical evaluation with endoscopy and/or esophageal pH monitoring, to confirm cases with functional heartburn which was beyond the scope of the current protocol [15]. This is also applicable for dyspepsia, requiring upper endoscopy to rule out organic causes and diagnosed functional dyspepsia [16]. Therefore, subjects fulfilling criteria for heartburn or dyspepsia were designated herein as uninvestigated heartburn and uninvestigated dyspepsia, respectively.

General questions regarding medical visits and medication use were included (have you consulted a physician for gastric, or intestinal problems? And, have you taken or are you currently taking any medication for your gastric or intestinal problems?). The Center for Epidemiological Studies Depression Scale (CES-D) [17] served as the instrument of psychological assessment. The CES-D is a 20 questions instrument commonly used to screen for symptoms of depression in the general population and has been validated in Mexico [18]. A total CES-D score from 0 to 14 is considered negative for depression, from 15 to 21 is considered mild to moderate depression, and a score higher than 22 is major depression [18]. Per standard, for the purpose of the current study, we used a threshold of ≥15 to identify subjects with depression.

The frequency of FGIDs is expressed in percentages with 95% Confidence Interval (95% CI). Categorical variables were analyzed by Chi-square and Fisher's exact test and continuous variables with the Students *t* test. A *P* value ≤ 0.05 was considered significant.

The protocol was approved by the local Institutional Review Board (IRB) for Health Research—2901—of the *Hospital General de Zona No. 1* of the *Instituto Mexicano del Seguro Social (IMSS)* in the State of Tlaxcala.

## 3. Results

The demographic characteristics of the study population are presented in Table 1. In the initial random sample of 500 households, 56 subjects were recruitment failures because of lack of availability or refusal and 10 because of inaccurate census information. In addition, five subjects were excluded due to medical reasons. In each case, a subject was selected from a neighboring household, per protocol. In the study population, two-thirds were women and almost a half were homemakers.

Criteria for at least one FGID were fulfilled by 292 subjects (58.4%) while 208 (41.6%) were without a FGID diagnosis herein designated as “controls”. The groups were similar in terms of age (mean ± SD): 40.3 ± 16.1 versus 39.5 ± 16.5 (*P* = 0.568); however, there were more women among those with FGIDs versus controls: 64.4% versus 56.2% (*P* = 0.039).  Table 2 depicts the general frequency of each FGID, including a summary by gender. The most common diagnoses were uninvestigated heartburn followed by IBS, functional abdominal bloating, unspecified functional bowel disorder, and FC. Interestingly, dyspepsia was relatively uncommon.

When comparing the group with FGIDs versus controls, subjects with *levator ani* syndrome (mean ± SD: 54.6 ± 28.8 years old) and fecal incontinence (49.7 ± 19.3), were significantly (*P* < 0.05) older than controls (40.4 ± 16.1). The prevalence of the majority of the FGIDs was similar between women and men except for IBS, IBS-C, IBS Alternating/Mixed (IBS-A/M), and functional abdominal bloating, which were significantly more common among women.

Importantly, the burden of health care utilization and psychological disease was increased in those with FGIDs. Subjects with FGIDs reported higher number of medical visits: 35.0 versus 17.0% (*P* < 0.05) and use of medication for gastrointestinal symptoms: 41.0 versus 22.0% (*P* < 0.05). In addition, depression was more frequent in the group with FGIDs compared to controls: 26.4% versus 6.7% (*P* < 0.001) Table 3. Further, depression was present in 37.6% of the subjects with FGIDs that consulted for medical care versus 20.4% that did not consult (*P* < 0.01).

## 4. Discussion

To the best of our knowledge this is the first population-based study to estimate the prevalence of the FGIDs in Mexico using the Rome II criteria. The FGIDs were common in the general population as nearly sixty percent fulfilled criteria for at least one FGID. The most common diagnoses were uninvestigated heartburn, IBS, functional abdominal bloating, unspecified functional bowel disorders, and FC. As postulated, IBS, IBS-C, and IBS-A/M were all significantly more frequent in women than men and there was a trend for FC. Notwithstanding, functional abdominal bloating was also more common in women. Finally, compared to controls, subjects with FGIDs were twofold more likely to seek medical consultations and to use medications for GI symptoms, while depression was four times more likely.

### 4.1. Uninvestigated Heartburn

In our population, one-fifth of the subjects fulfilled criteria for heartburn. This finding is in agreement with those from other population-based studies that have reported a high frequency of GER-related symptoms. For example in Spain, in a telephone-based survey, Diaz-Rubio et al. found that 32% of the subjects reported GER symptoms [19]. In our study the diagnosis of heartburn was based only on symptom reporting with the RIIQ without additional diagnostic investigation, therefore the true prevalence of functional heartburn cannot be estimated. In a previous study in Mexico in patients fulfilling criteria for heartburn according to the RIIQ, 62.0% had GER confirmed by endoscopy and/or pH monitoring [20]; this study was limited by the fact that pH impedance testing was not used. Notwithstanding, based on those results, we may assume that of the 98 subjects that fulfilled criteria for heartburn in the present study, 61 (62.0%) will probably have true GER and 37 (38.0%) may have functional heartburn, thus estimating a prevalence of 7.4% (37/500) for functional heartburn in our population. This result is in agreement with a population-based study from Australia using Rome II criteria, reporting a prevalence of functional heartburn of 10.4% [21].

### 4.2. Uninvestigated Dyspepsia

The low frequency (7.0%) of dyspepsia is an interesting finding, which may reflect a true low-population prevalence of functional dyspepsia in Mexico and/or it may be related with aspects of the RIIQ in the assessment of functional dyspepsia. Similar to uninvestigated heartburn, subjects did not undergo endoscopy to rule out organic etiologies. Thus, we suggest that the prevalence reported herein corresponds to uninvestigated dyspepsia. This result is in accordance with our previous study among volunteers in Mexico City with the RIIQ, where dyspepsia was present in 8.0% of the subjects [8]. Furthermore, a similar study from Canada with the RIIQ found a very low prevalence of dyspepsia (1.8%) [22]. This contrasts with a study from Brazil that used modified Rome II criteria and found a 48% frequency of uninvestigated dyspepsia [23]. These observations suggest that in Mexico, dyspepsia is uncommon compared to other FGIDs such as IBS, understanding that the Rome II criteria may have inherent limitations with respect to the diagnosis of functional dyspepsia.

### 4.3. Irritable Bowel Syndrome

Globally, IBS is considered the most frequent FGID with a prevalence that ranges from 5 to 25% [1, 22]. This variability is probably related to the use of different diagnostic criteria between the studies, study designed differences (e.g., convenience samples versus population-based sampling), as well as true population differences. In fact, a recent joint conference of the Rome Foundation and the World Gastroenterology Organization (WGO) about the global perspective of IBS concluded that it was necessary to conduct population-based studies to estimate the frequency of this functional bowel disorder worldwide [24]. The 16.0% frequency of IBS in the current study is concordant with a parallel study that was conducted in Central America (Nicaragua) using Rome II criteria that reported a 13.2% prevalence [25] and with the 19.9% reported in a population-based study from South America (Colombia), using Rome III criteria [26]. In contrast, our prevalence is eight times higher than the one reported in a multinational study in Europe using Rome II criteria [1]. In that study, dyspepsia was also more common than IBS, with a prevalence that ranged from 15.1% to 23.9% [27, 28]. In the current study, IBS proved to be more frequent in women than men and in the IBS subtypes, IBS-C, and IBS-A/M. This gender difference did not hold up for IBS-D as has been reported in other studies [29]. Further, in this population-based study IBS-A/M is the most frequent subtype followed by IBS-C and IBS-D. This is consistent with our prior studies in Mexico [8, 9]. The higher frequency of IBS-C compared to IBS-D seems to be a common finding in Latin American studies [26, 30], except for Argentina [31] where IBS-D is the predominant subtype. This latter discordance might relate to genetic and environmental influences in populations such as Argentina with a greater European influence. Also, in one of the first cross-cultural studies of IBS conducted in the USA, Mexico, Canada, England, Italy, Israel, India, and China, using the Bowel Symptom Scale (BSS), diarrhea was less frequent than constipation [32]. The Mexican subjects reported the highest score for constipation while those from China reported the highest score for diarrhea [32]. The higher frequency of IBS-D in China was confirmed in a recent study among patients with IBS using the Rome II criteria, in which 65.9% was diagnosed as IBS-D while 26.4% was diagnosed as IBS-C [33]. The contrasts between the different studies underscore the importance of diagnostic criteria, study methodology, and subject populations, all important factors than can influence the reported frequencies of IBS and the IBS subtypes. Use of standard criteria and methodology is imperative in future studies to elucidate the worldwide frequency of IBS.

### 4.4. Functional Constipation

A recent systematic review and meta-analysis of population-based studies from around the world, with limited data from Latin America, reported a pooled prevalence of 14% (95% CI: 12–17) for FC. The prevalence of FC was lower in South East Asian studies and in those using the Rome II or III criteria [34]. The lower prevalence found in our study using the Rome II criteria [7.4% (5.3–10.1)] is concordant. In contrast, a recent meta-analysis that included the results from the current survey and those from other available studies in Mexico reported a pooled prevalence of FC of 14.4% (12.6–16.6). Although this meta-analysis found similar figures to those from the first systematic review [35], they are higher than the ones from the current survey, probably influenced by the inclusion of data from studies conducted in convenience samples in Mexico contrary to the current one using a population-based sampling strategy.

### 4.5. The Burden of FGIDs: Health Care Utilization and Depression

Psychological comorbidities such as anxiety and depression have been associated with FGIDs. For example, depression has been associated with GER symptoms [36, 37]; patients with IBS and functional dyspepsia [38]; IBS-C with higher symptoms severity [39, 40]; IBS with lower HRQOL [39, 40]. Although psychological comorbidities are frequent among FGID subjects that seek medical care, few studies have analyzed such associations in subjects with FGIDs from the community [41]. Furthermore, subjects with depression in the community report more frequently gastrointestinal symptoms such as abdominal pain, diarrhea, constipation, dyspepsia, and/or IBS [42]. In the current study, we used a validated instrument to screen for depression, and we confirmed that depression is four times more likely to be present among subjects with FGIDs than those without a FGID and in those that consulted compared to those who did not. This finding is in agreement with a study in primary care showing that severe depression was five times more likely among subjects with gastrointestinal symptoms [43]. With regard to IBS, we found that 47.5% of our subjects reported depression. This is consistent with a previous study among patients that consulted a referral center in Mexico, in which 46.0% had trait depression according to the Hospital Anxiety and Depression Scale (HAD) [44]. In summary, these results suggest that depression in subjects with FGIDs in the community is common and is more frequent among those that seek medical care.

The high frequency of FGIDs in the general population is remarkable and suggests that having at least one FGID is “normal”. This finding is in agreement with data from a study that followed subjects for over 20 years in Olmsted County Minnesota and reported that 89% fulfilled criteria for at least one FGID [45]. Also, in this population-based cohort, health care utilization was increased in FGIDs and IBS subjects. One-third of the subjects with FGIDs had related medical care, thus they can be considered “patients”. Among those with IBS, 56.2% had recent medical visits for GI symptoms, thereby also considered “patients”. Our results are similar to those from other parts of the world [46]. In addition, almost 70% of the IBS subjects had used medications for their symptoms. These findings provide an indirect estimation of the IBS burden of illness in Mexico.

Our study has several limitations. It was conducted in a single State in Mexico; however, we consider that it is representative of the mestizo population which predominates in this country [47]. Secondly, we did not include an assessment of socioeconomic status or its possible relation to the FGIDs, nor to the consultation behavior. Third, while we did not screen for anxiety, we used a validated depression instrument, thereby strengthening our results. Lastly, the survey was conducted using the Rome II and not the more recent Rome III criteria. However, a Rome III-based epidemiological study is underway in Mexico and Central America and these results will allow us to further elucidate the FGIDs prevalence and potential instrument differences [48].

In conclusion, in this population-based study in Mexico, FGIDs and IBS are observed to be common with important gender differences for IBS and functional abdominal bloating. Among subjects with FGIDs and/or IBS, health care utilization is increased and a positive association with depression is observed. Further, studies of FGIDs epidemiology in Mexico and Latin America are warranted.

## Figures and Tables

**Table 1:** Demographic characteristics of the study population.

Item	Study population	95% CI
*Age*: (mean ± SD)	39.8 ± 16.3	38.7–41.2
*Sex*: *n* (%)		
(i) Women	305 (61.0)	56.6–65.3
(ii) Men	195 (39.0)	34.7–43.4
*Occupation*: *n* (%)		
(i) Homemaker	220 (44.0)	39.6–48.5
(ii) Employee	115 (23.0)	19.4–26.9
(iii) Self-employed	100 (20.0)	16.6–23.8
(iv) Student	30 (6.0)	4.1–8.5
(v) Manual labor	20 (4.0)	2.5–6.1
(vi) Other activities	15 (3.0)	1.7–4.9
*Marital Status*: *n* (%)		
(i) Married	303 (60.6)	56.2–64.9
(ii) Single	111 (22.2)	18.6–26.1
(iii) Civil Union	43 (8.6)	6.3–11.4
(iv) Widower	24 (4.8)	3.1–7.1
(v) Separated/Divorced	19 (3.8)	2.3–5.9

**Table 2:** Prevalence of functional gastrointestinal disorders.

FGID	All (*n* = 500)		Women (*n* = 305)	Men (*n* = 195)	*P* (women versus men)
	*n*	% (95% CI)	*n* (%)	*n* (%)	
	292	58.4 (53.9–62.8)	188 (61.6)	104 (53.5)	0.066

*Esophageal disorders*					

Globus	9	1.8 (0.8–3.4)	6 (2.0)	3 (1.5)	0.725
Rumination syndrome	4	0.8 (0.2–2.0)	2 (0.7)	2 (1.0)	0.651
Functional chest pain of presumed esophageal origin	15	3.0 (1.7–4.9)	11 (3.6)	4 (2.1)	0.320
Uninvestigated heartburn	98	19.6 (16.2–23.4)	57 (18.7)	41 (21.0)	0.521
Dysphagia	9	1.8 (0.8–3.4)	5 (1.6)	4 (2.1)	0.735

*Gastroduodenal disorders*					

Uninvestigated dyspepsia	35	7.0 (4.9–9.6)	23 (7.5)	12 (6.2)	0.553
(i) Ulcer-like	17	3.4 (2.0–5.4)	12 (3.9)	5 (2.6)	0.410
(ii) Dysmotility-like	18	3.6 (2.1–5.6)	11 (3.6)	7 (3.6)	0.992
Aerophagia	28	5.6 (3.8–8.0)	15 (4.9)	13 (6.7)	0.399
Functional vomiting	10	2.0 (1.0–3.6)	8 (2.6)	2 (1.0)	0.213

*Bowel disorders*					

IBS	80	16.0 (12.9–19.5)	62 (20.3)	18 (9.2)	**0.001**
(i) IBS-D	12	2.4 (1.2–4.2)	7 (2.3)	5 (2.6)	0.841
(ii) IBS-C	33	6.6 (4.6–9.1)	29 (9.5)	4 (2.1)	**0.001**
(iii) IBS-A/M	45	7.0 (4.9–9.6)	26 (8.5)	9 (4.6)	**0.003**
Functional abdominal bloating	54	10.8 (8.2–13.9)	41 (13.4)	13 (6.7)	**0.017**
Functional constipation	37	7.4 (5.3–10.1)	28 (9.2)	9 (4.6)	0.057
Functional diarrhea	7	1.4 (0.6–2.9)	3 (1.0)	4 (2.1)	0.322
Unspecified eunctional bowel disorder	53	10.6 (8.0–13.6)	30 (9.8)	23 (11.8)	0.488

*Functional abdominal pain*					

Functional abdominal pain syndrome	5	1.0 (0.3–2.3)	4 (1.3)	1 (0.5)	0.381
Unspecified functional abdominal pain	8	1.6 (0.7–3.1)	6 (2.0)	2 (1.0)	0.413

*Biliary disorders*					

Gallbladder dysfunction	6	1.2 (0.4–2.6)	5 (1.6)	1 (0.5)	0.259
Sphincter of oddi dysfunction	1	0.2 (0-1.1)	1 (0.3)	0 (0.0)	0.423

*Anorectal disorders*					

Functional fecal incontinence	23	4.6 (2.9–6.8)	13 (4.3)	10 (5.1)	0.652
(i) Soiling	14	2.8 (1.5–4.7)	7 (2.3)	7 (3.6)	0.392
(ii) Gross incontinence	9	1.8 (0.8–3.4)	6 (2.0)	3 (1.5)	0.725
Levator ani syndrome	7	1.4 (0.6–2.9)	3 (1.0)	4 (2.1)	0.322
Proctalgia fugax	31	6.2 (4.3–8.7)	22 (7.2)	9 (4.6)	0.240
Dyssynergia	10	2.0 (1.0–3.6)	8 (2.6)	2 (1.0)	0.213

IBS: irritable bowel syndrome, IBS-D: irritable bowel syndrome diarrhea predominant, IBS-C: irritable bowel syndrome constipation predominant, IBS-A/M: irritable bowel syndrome alternating/mixed. There were no differences in the prevalence of the different FGIDs between women versus men, except for IBS IBS-C IBS-A/M and functional abdominal bloating that were all more frequent among women and a trend for functional constipation.

**Table 3:** Depression, medical visits, and use of medications by FGID.

Diagnosis		Age	Women	Depression (CES-D)	Medical visits for GI problems	Use of medications for GI problems
	*n*	(mean ± SD)	(%)	(%)	(%)	(%)
*Esophageal disorders*						

Globus	9	40.4 ± 12.0	66.7	11.1	11.1	44.4
Rumination syndrome	4	46.0 ± 11.2	50.0	75.0	50.0	50.0
Functional chest pain of presumed esophageal origin	15	39.4 ± 14.9	73.3	46.7	33.3	60.0
Uninvestigated heartburn	98	37.2 ± 13.8	58.2	26.5	45.9	44.9
Dysphagia	9	50.3 ± 17.9	55.5	44.4	55.6	55.6

*Gastroduodenal disorders*						

Uninvestigated dyspepsia	35	37.6 ± 14.7	65.7	17.1	25.7	40.0
(i) Ulcer-like	17	40.1 ± 18.3	70.6	17.6	23.5	41.2
(ii) Dysmotility-like	18	35.2 ± 10.4	61.1	16.7	27.8	38.9
Aerophagia	28	41.7 ± 19.7	53.6	50.0	39.3	53.6
Functional vomiting	10	32.2 ± 12.7	80.0	30.0	70.0	80.0

*Bowel disorders*						

IBS	80	40.4 ± 17.5	77.5	47.5	56.2	67.5
(i) IBS-D	12	43.3 ± 20.8	58.3	50.0	50.0	66.7
(ii) IBS-C	33	40.7 ± 20.1	87.9	60.6	66.7	69.7
(iii) IBS-A/M	45	39.3 ± 13.6	74.3	40.0	48.6	65.7
Functional abdominal bloating	54	37.0 ± 16.2	75.9	24.1	40.7	42.6
Functional constipation	37	37.6 ± 18.7	75.7	24.3	35.1	37.8
Functional diarrhea	7	42.3 ± 24.2	42.9	42.9	42.9	28.6
Unspecified functional bowel disorder	53	38.5 ± 15.9	56.6	13.2	17.0	26.4

*Functional abdominal pain*						

Functional abdominal pain syndrome	5	38.2 ± 9.6	80.0	100.0	60.0	60.0
Unspecified functional abdominal pain	8	35.4 ± 8.7	75.0	25.0	75.0	50.0

*Biliary disorders*						

Gallbladder dysfunction	6	27.7 ± 8.8	83.3	50.0	66.7	83.3
Sphincter of oddi dysfunction	1	27.0	100.0	0	0	0

*Anorectal disorders*						

Functional fecal incontinence	23	49.6 ± 19.2	56.5	52.2	65.2	60.9
(i) Soiling	14	45.4 ± 15.9	50.0	50.0	71.4	57.1
(ii) Gross incontinence	9	56.2 ± 23.0	66.7	55.6	55.6	66.7
Levator ani syndrome	7	54.6 ± 28.8	42.9	71.4	71.4	71.4
Proctalgia fugax	31	41.4 ± 16.5	71.0	61.3	45.2	54.8
Dyssynergia	10	42.3 ± 19.9	80.0	60.0	30.0	40.0

CES-D: center for epidemiological studies depression scale, GI: gastrointestinal, IBS: irritable bowel syndrome, IBS-D: irritable bowel syndrome diarrhea predominant, IBS-C: irritable bowel syndrome constipation predominant, IBS-A/M: irritable bowel syndrome alternating/mixed.
